# Cloning of full genome sequence of hepatitis E virus of Shanghai swine isolate using RACE method

**DOI:** 10.1186/1743-422X-4-98

**Published:** 2007-10-09

**Authors:** Quan Shen, Wen Zhang, Xiangrong Cao, Jing Mou, Li Cui, Xiuguo Hua

**Affiliations:** 1 School of Agriculture and Biology, Shanghai JiaoTong University, 800 Dongchuan Road, Shanghai 200240, PR China; 2 School of Life Science, Nanjing Normal University, 1 Wenyuan Road, Nanjing 210097, PR China

## Abstract

Genotype 4 hepatitis E virus (HEV) was reportedly transmitted freely between humans and swine in eastern China. The full-length genomic sequence of Shanghai swine isolate (SH-SW-zs1) recovered from feces sample of a pig which was infected with HEV RNA positive swine serum was determined using RT-PCR and RACE (Rapid Amplification of cDNA Ends) methods. The full genome of the SH-SW-zs1 isolate was 7265 nucleotides in length and phylogenetic analysis indicated that this isolate belonged to genotype 4. Comparison of the 3' UTR sequence with the corresponding regions of other 38 HEV strains from different region revealed that the Shanghai swine isolate is 21–49 bp longer than the other stains.

## Introduction

Hepatitis E is an important public health disease in many developing countries of Asia and Africa and also occurs sporadically in some industrialized countries [1-4]. The disease mainly affects young adults and has a relatively high mortality of up to 25% in affected pregnant women [1]. The main mode of transmission of hepatitis E virus (HEV) is fecal-oral route, primarily through contaminated water supplies [1]. HEV is single-stranded, positive-sense RNA virus without an envelope [5]. The genome of HEV is approximately 7.2 Kb and consists three open reading frames (ORF1–3) [6]. ORF1 locates at the 5 ' end of genome and encodes non-structural proteins, including the methyltransferase, protease, helicase and RNA-dependent RNA polymerase (RdRp) [7]. ORF2 maps to the 3 ' terminus and encodes for a major structural protein, and ORF3 overlaps both and encodes a thus far unknown function [6]. Based on sequence analysis, HEV sequences have been classified into four major genotypes (1–4). Genotype 1 is the main cause of hepatitis E in developing countries in Asia and Africa, and genotype 2 has been documented in Mexico and Nigeria. Genotype 3 or 4 have been described in the United States, European countries, China, Taiwan, and Japan [8,9]. The virus is also prevalent in swine, and isolates from swine are genetically closely related to that from humans [10-12]. Lots of researches showed that genotype 4 and genotype 1 were the major genotype in China, recently genotype 3 HEV was reported in swine of Shanghai suburb [13]. For the further research, such as genomic characteristics and phylogenetic analysis, the full genome of the isolate which was proved prevalent in Shanghai swine was determined in the current study.

## Materials and methods

### Samples

132 serum samples of swine were collected from Shanghai suburb in China. These samples were tested for HEV RNA, using reverse transcriptase-polymerase chain reaction (RT-PCR). One HEV RNA positive swine serum sample was used for experimental infection of pigs [14]. HEV RNA positive swine fecal samples were stored as 10% suspension in aliquots at 70°C. About 10 g of HEV RNA positive fecal sample was converted to 10% (w/v) suspensions in PBS (0.01 M, pH 7.2–7.4, added 0.1% DEPC) for determining the full genomic sequence of HEV.

### Viral RNA extraction

One hundred microlitre of fecal suspensions was mixed with 1 ml of trizol (invitrogen, USA). The mixture was homogenized and incubated for 5 min at room temperature. Two hundred microlitre of chloroform was added and the mixture was vigorously shaken for 15 s and incubated at room temperature for 3 min. The aqueous phase was transferred to a fresh microfuge tube after centrifugation at 12 000 g for 15 min at 4°C. Five hundred microlitre of isopropyl was added and the mixture was incubated for 15 min at room temperatures. Then centrifuging at 12 000 g at 4°C for 15 min. After discarding the supernatant, RNA pellet was washed with 1 ml 75% ethanol. The RNA pellet was Dried at room temperature for 5 min after centrifuging at 5 000 g for 5 min at 4°C and Discarding the supernatant. RNA sample was dissolved with 20 ul DEPC-treated water and used to reverse transcription immediately.

### PCR amplification

Full-length primers: 18 sets of degenerate primers were designed based on a multiple sequence alignment of entire genome from isolates AY594199, DQ279091, DQ450072 and AB108537 (table 1). Reverse transcription was carried out at 42°C for 1 h with 1 ul (200 units) of AMV Reverse Transcriptase (TakaRa, Japan) and 1 ul (25 mM) of external antisense primer. The first round PCR was carried using 10 ul of the synthesized cDNA and an external set of forward and reverse primers with Ex Taq DNA polymerase (TakaRa, Japan). A nested PCR was carried out with internal primer set and 5 ul of the first PCR product. The PCR parameters of all amplification reactions included an initial incubation at 95°C for 9 min, followed by 39 cycles of denaturation at 94°C for 1 min, annealing for 1 min at a temperature varied according to the Tm of different primers, and extension at 72°C for 1.5 min, with a final incubation at 72°C for 7 min. The resulting PCR products were excised from agarose gel and purified using the Axyprep DNA Gel Extraction Kit (AXYGEN, USA). The purified PCR products were ligated into PMD18-T vector (TakaRa, Japan) using T4 DNA ligase (TakaRa, Japan) at 16°C overnight. The recombinant plasmid was transformed into DH5α competent Escherichia coli cells (TakaRa, Japan). Plasmids containing the insert fragment were identified by PCR. Three of the positive clones were sequenced.

**Table 1:** 

Primer name	Nucleotide position	Nucleotide sequence (5'-3')
HE0ES	104-84	CGGAGTTGGCCGCTGCTAGAG
HE0EA	482–501	TGTACT(G)TTTGCTGCTGAGAC
HE0IS	225-203	ATTGGGTGATTCCACAG(A)AACCTC
HE0IA	236–256	ATCCACAAC(T)GAGCTT(C)GAGCAG
HE1ES	11–32	TATGTGGTCGACGCCATGGAGG
HE1EA	528-509	GCCCTTTATTCACTGCACGA
HE1IA	573-554	ATACCGTGGCGAGCCATTGC
HE2ES	482–501	TGTACTTTTGCTGCTGAGAC
HE2EA	956–975	ACAGGGACGGCATGAAATGT
HE2IS	437–454	CTTCCACCTGT(C)T(C)GAT(C)CGG
HE2IA	1000-983*s*	AAGCATA(G)AGCCTGTCCCA
HE3ES	671–692	CGTGCA(T)GTG(A)ATTACATAT(C)GAGG
HE3EA	1336-1317	CCACCGG(T)CGAA(G)CACTGG(A)GCAT
HE3IS	742–762	GATCCGT(G)ACC(G)ACT(C)AAGGTCAC
HE3IA	1314-1293	AACTG(C)CAA(G)CTGA(G)CGA(G)CCAGGGA
HE4ES	984–1005	GGGACAGGCTTATGCTTTTTGG
HE4EA	1528-1508	TGCCTCATTATCATAACCCTG
HE4IS	956-975	ACGTTTCATGCCGTCCTGT
HE4IA	1703-1684	GGCCGTCG(A)GCA(G)TCAGAG(A)ACC(T)
HE5ES	1331-1348	CGGTGGT(C)TG(A)TCTGCC(T)GGC
HE5EA	1792-1746	GTTGAG(A)AAGGTT(C)TTATTG(A)
HE5IS	1310–1329	C(G)AGTTT(C)TATGCCCAGTGTCG
HE5IA	1803-1785	GACAG(A)C(G)ACATAC(T)TGCTCT(C)G
HE6ES	1508–1528	CAGGGT(C)TATGAT(C)AAT(C)GAGGC
HE6EA	2529-2510	GGGAAC(A)CGT(C)TGA(G)TAGAAT(A)GC
HE6IS	1679–1700	GTTGAG(A)GTC(T)TCTGAT(C)GCC(T)GACG
HE6IA	2477-2457	GGTTA(G)GAT(C)GCATTA(G)ACCAGCC
HE7ES	2028–2048	TGTGGTAC(T)T(C)AC(T)CCTGAGGGGC
HE7EA	2144-2123	CTCTACACT(C)CGG(T)ACCTGGTCGG
HE7IS*	2830–2850	GTAAGGGCTGGAAGGGTGGGC
HE7IA*	2913-2893	ACTTCAGTGGCGGAGTCTAAC
HE8ES	2753–2772	GCCTGGGAACGTAACCACCG
HE8EA	3366-3347	GTCTGGATC(T)TTT(C)GGGTACGC
HE8IS	2714–2733	GCCGGC(T)ATATATAAGGTC(A)CC
HE8IA	3438-3416	GCCTGGGTG(A)AAT(C)ACCAA(G)CTTCT(C)G
HE9ES	3209–3228	GGTGAC(T)CCC(T)AAT(C)AAT(C)AAATCCC
HE9EA	3948-3929	GGCGCTGCCATACGGCAGTG
HE9IS	3312–3334	GATGC(T)CCGGCG(A)GAT(C)GTCTGTGAG
HE9IA	3810-3791	GGTCGA(G)TGGCCAAGC(T)TCCTC
HE10ES	3764–3781	CAGTTTAGTGCT(C)TAC(T)CAG
HE10EA	4432-4413	ATCATTCTCAAAAACCTTAC
HE10IS	3587–3605	ACG(T)GAGAAG(A)TGTGTGGTG(C)G
HE10IA	4518-4496	CACTCC(T)TCCATGATTATACACTC
HE11ES	4290–4311	TGTTC(T)GGCCCA(C)TGGTTT(C)CGCGC
HE11EA	4752-4733	CGATAGTCACTACAGAGCAC
HE11IS	4355–4375	TATGGTGATGCA(G)TATGAG(A)GAC
HE11IA	4736-4717	GCACAACAGAATCATCTCCC
HE12ES	4607–4625	TGGAAGAAA(G)CAT(C)TCTGGTG
HE12EA	5253-5233	CCGGTGGCGCGGGCAGCATAG
HE12IS	4496–4518	GAGTGTATAATCATGGAG(A)GAGTG
HE12IA	5347–5366	GGTTGGATGAATATAGGGGA
HE13ES	4977-4997	CGAATGTGGCTCAGGTTTGTG
HE13EA	5451-5431	GCCAAGCGGAACCGAGTGGAC
HE13IS	5020–5039	CGGTGTTAGCCCTGGCTTGG
HE13IA	5392-5371	GTTGGAATGTCGGATGCGAAGG
HE14ES	5347–5366	TCCCCTATATTCATCCAACC
HE14EA	5956-5934	TGATTG(T)CGATAG(A)TGCAGGCGCTC
HE14IS	5233–5252	CTATGCTGCCCGCGCCACCG
HE14IA	5980-5957	GAGGTCTCAACT(C)GAG(A)CGCCAA(G)CCC
HE15ES	5922–5942	GTGATT(C)CCTAGT(C)GAGCGCCTG
HE15EA	6415-6397	GTCGGCTCGCCATTGGCTG
HE15IS	5877–5896	ACTGATGTCCGC(G)ATC(T)CTTGT
HE15IA	6453-6433	CCTGCTGAGCATTCTCGACTG
HE16ES	6336–6357	CTC(A)CCGACAGAATTGATTTCGT
HE16EA	7005-6985	CAGAG(A)TGA(G)GGT(G)GCA(G)AGGACAC
HE16IS	6271–6292	TTGGTGAG(A)GTT(C)GGC(T)CGTGGTAT
HE16IA	7074-7054	CAGGGCAA(G)AG(A)ATCATCG(A)AAAG
HE17ES*	6763–6782	CGCTCACTACTATCCAGCAG
HE17IS*	6787–6808	CTAAGACCTTCTTTGTTCTGCC
HE17A		GTTTTCCCAGTCACGACTTTTTTTTTTTTTTT
*: the primers were designed according to isolate in this study.		

Position and nucleotide sequence of oligonucleotide primers for PCR. The nucleotide position is in accordance with the SH-SW-zs1 isolate in this study. In the primer name, ES, EA, IS and IA mean "external sense", "external antisense", "internal sense" and "internal antisense", respectively. Letters in parentheses indicate degenerate bases.

### 5'RACE

The 5'RACE was carried out with the 5-Full RACE Core Set (TaKaRa, Japan) kit following the manufacture's instructions. Briefly, 1st strand cDNA was Synthesized by reverse transcription using 5'end-phosphorylated RT Primer which was specific to the swine HEV (5'-p-GTCATRCCRTGGCG-3'). The PCR reaction mixture was incubated for 2 min at 94°C followed by 35 amplification cycles, comprising denaturation at 94°C for 30 s, annealing at 65°C for 30 s and extension at 72°C for 30 s. The reaction was extended for another 7 min at 72°C to insure the full extension. Fifteen ul of 1st Strand cDNA was treated with RNase H in a total 75 μl reaction mixture containing 15 ul of Hybrid RNA Degeneration Buffer for 1 h at 30°C. The mixture was then precipitated at -20°C for 30 min, being added 100 ul of H_2_O and 500 ul 100% ethanol. The supernatant was discarded and the pellet was washed with 75% ethanol after centrifuging at 12 000 g for 5 min. The pellet was dissolved with 8 ul of RNA (ssDNA) Ligation Buffer and 12 ul of H_2_O after dried at room temperature for 5 min. 20 ul of 40% PEG-6000 and 1 ul of ligase were added and incubated at 16°C overnight. Fifteen microliters of circled cDNA was then used as template for nested PCR using ExTaq DNA polymerase (TaKaRa, Japan)with two sets of primers: 5'-CGGAGTTGGCCGCTGCTAGAG-3'(external forward primer, nucleotide position numbers 104 to 84), 5'-TGTACT(G)TTTGCTGCTGAGAC-3'(external reverse primer, nucleotide position numbers 482 to 501), 5'-ATTGGGTGATTCCACAG(A)AACCTC-3'(internal forward primer, nucleotide position numbers 225 to 203), and 5'-ATCCACAAC(T)GAGCTT(C)GAGCAG-3'(internal reverse primer, nucleotide position numbers 236 to 256). The PCR reaction mixture was incubated for 2 min at 94°C followed by 35 amplification cycles, comprising denaturation at 94°C for 30 s, annealing at 65°C for 30 s and extension at 72°C for 30 s. The reaction was extended for another 7 min at 72°C to insure the full extension. The final PCR product was analyzed on 20 g/L agarose gel.

### 3'RACE

The 3'RACE was carried out with the TaKaRa RNA PCR Kit (TaKaRa, japan) following the manufacture's instructions. Brifely, ten microliters of the HEV RNA was used as template to synthesize cDNA with AMV Reverse transcriptase for 1 h at 42°C. The external reverse primer (HE17A) which has a poly (T) tract was used to prime the cDNA synthesis. The cDNA was then amplified by nested PCR with the external forward primer (5'-CGCTCACTACTATCCAGCAG-3', nucleotide position numbers 6763–6782) and internal forward primer (5'-CTAAGACCTTCTTTGTTCTGCC-3', nucleotide position numbers 6787–6808) with ExTaq DNA polymerase (TaKaRa, Japan). The PCR reaction mixture was incubated for 2 min at 94°C, followed by 35 amplification cycles comprising denaturation at 94°C for 30 s, annealing at 65°C for 30 s, and extension at 72°C for 30 s. The reaction was extended for another 7 min at 72°C to ensure the full extension.

### Phylogenetic analysis

Using Clustal × 1.8, multiple alignments of nucleotide sequences was carried out. The phylogenetic status SH-SW-zs1 isolate was assessed employing the software MEGA Version 2.1[15]. For analysis in MEGA, Jukescantor (JC) distance was utilized employing the Neighbor joining (NJ) algorithm. The reliability of different phylogenetic groupings was evaluated by using the bootstrap test (1000 bootstrap replication) available in MEGA. Accession numbers, designations and countries of origin of the full genome sequences employed for analysis in the present study were as follows:

Genotype 1: AF051830, Nepal; X99441, India; AF076239, India; AF459438, India; D10330, Burma; M73218, Burma; AF185822, Pakistan; X98292, India; L25595, China; M80581, Pakistan; AY230202, Morocco.

Genotype 2: M74506, Mexico.

Genotype 3: AP003430, Japan, human; AB091394, Japan, human; AB073912, Japan, swine; AY115488, Canada, swine; AF060668, US, human; AF082843, US, swine; AB089824, Japan, human; AB074918, Japan, human; AB074920, Japan, human.

Genotype 4: AB091395, Japan, human; AB097812, Japan, human; AB097811, Japan, swine; AB074915, Japan, human; AB074917, Japan, human; AJ272108, China, human; AB108537, China, human; AB161717, Japan, human; AB161718, Japan, human; AB161719, human; DQ450072, China, swine; AY594199, China, swine; DQ279091, China, swine; AB197673, China, human; EF077630, China, swine; AB197674, human.

Avian Hepatitis E virus (AY535004) was chosen as an out-group. The sequence reported here has been deposited with GenBank accession no.: EF570133.

## Results

### 3'RACE

**Figure 1:**
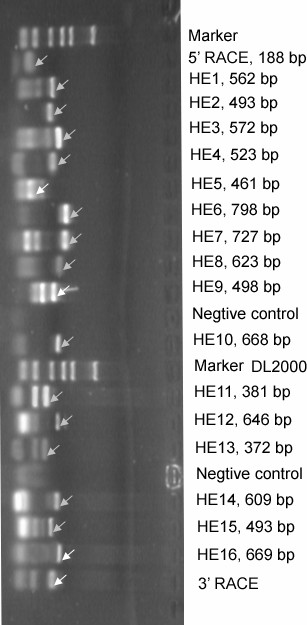
RT-PCR products of SH-SW-zs1 isolate. The right side shows the primers and the expected length of the fragment; Arrows display the aimed bands.

As shown in Figure 1, 3'RACE band of the expected size was obtained. The 3' terminus of this study had 93 nucleotides upstream of the polyA. The sequence of 3'UTR was: TTT ATT CTT CTT GTA CCT CCC CTT CGG TTC TGT TTC TTT TTA TTT CTC CTT TCT GCG TTC CGC GCT CAC TAC TAT CCA GCA GGA TCC ATG TTG. Comparison of the 3'UTR sequence with the corresponding regions of other 38 HEV strains from different region of the world revealed that the Shanghai swine isolate is 21–49 bp longer than all the other stains (additional file).

### Analysis of Full-Length Genome of Shanghai Isolate

**Figure 2:**
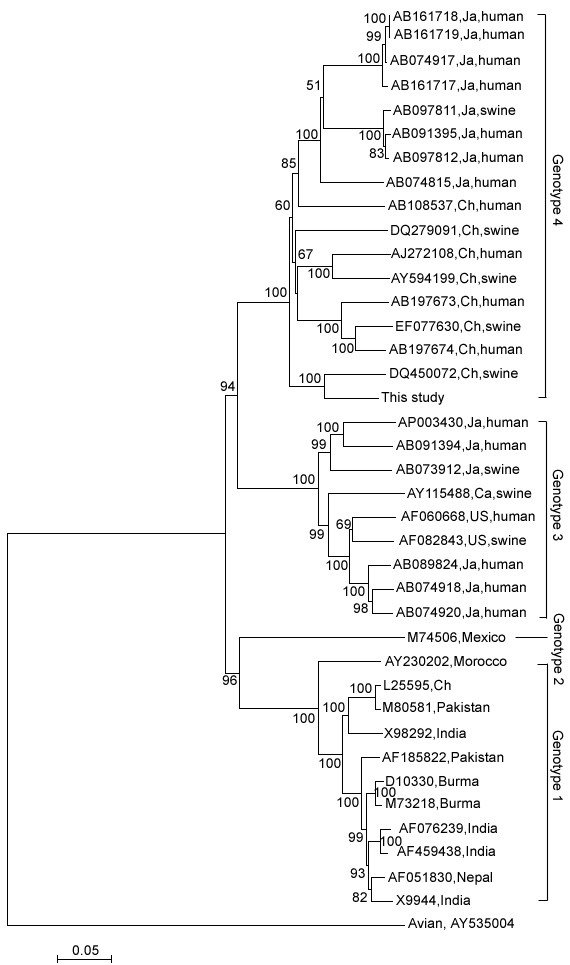
Phylogenetic trees constructed using MEGA software depicting genotypic status of SH-SW-zs1 on the basis of full-length genome sequence of 39 HEV isolates. Genbank accession numbers for the full genome were marked at each branch. Percent bootstrap support is indicated at each node. The abbreviations Ch and Ja stand for China and Japan, respectively.

The genomic length of the SH-SW-zs1 isolate was determined to be 7265 nucleotides (nt) excluding poly (A) tail at 3' terminus and contained three open reading frames (ORFs) similar to earlier reported human and swine HEV isolates. The genomic organization consisted of 5' untranslated region (5'UTR) of 25 nt (1–25), ORF-1 of 5127 nt (26–5152), ORF-2 of 1983 nt (5190–7172), ORF-3 of 372 nt (5249–5520) and 3'UTR of 93 nt (7173–7265), followed by a poly (A) tail of 26 residues. The length of 5'UTR was same as that of other type 4 isolates and had nucleotide G at the extreme 5' end of the genome as other reported genotype 4 sequences. Whole genome-based phylogenetic analysis confirmed classification of Shanghai swine in genotype 4 (Fig. 2). The phylogenetic tree showed that genotype 4 could be divided into 3 main subgroups. SH-SW-zs1 isolate closely clustered with isolate DQ450072 which was isolated from eastern China, and they shared 89.3% identity (with divergence of 11.3%) with each other and represented a distinct subgroup among the genotype 4 isolates with a bootstrap value of 100%.

## Discussion

HEV is the major cause of enterically transmitted non-A, non-B, non-C hepatitis and is responsible for significant morbidity and mortality in developing countries [16]. Outbreaks of hepatitis E have been described in Asia, Africa and Mexico [16-18], while sporadic cases have been reported in the United States, Japan and other developed countries [8]. It has been shown that HEV is a zoonotic virus [19,20]. Hitherto, the lack of an efficient cell-culture system for HEV has greatly hampered detailed analysis of the virus replication cycle in infected cells, which makes it difficult to resolve many important questions. Meanwhile, cloning full-length genome of HEV is an efficient way to analysis molecular character, viral replication and other problems. Some reports indicated that genotype 4 and genotype 1 were the major genotype in China, though genotype 3 HEV was recently found in swine of Shanghai suburb [13]. Recent observations suggested that the HEV genotype influences the severity of hepatitis E, and that genotype 4 is associated more strongly with the severe form of hepatitis E than genotype 3 [21]. Therefore, the genomic full-length of the Shanghai isolate was determined in this study for further demonstrating the HEV strain prevalent in eastern China. The full genome of the SH-SW-zs1 isolate was 7265 nucleotides in length and phylogenetic analysis indicated that this isolate belonged to genotype 4. This isolate closely clustered with isolate DQ450072 and they shared 89.3% identity(with divergence of 11.3%) with each other and represented a distinct subgroup among the genotype 4 isolates with a bootstrap value of 100%, thus suggested that they may come from one common strain. Result of comparison showed that the 3'UTR of this Shanghai isolate was 21–49 bp longer than all the other stains so far avalible on-line. By blast the 21-nt-fragment in GenBank, we found it has many homologous sequences which shared more than 85% identity with it. So we presumed that this fragment may come from the recombination of genome HEV and its host or other microorganism. The true origin of this short fragment and its specific function need to be further studied.

## Supplementary Material

Additional file 1 Comparison of length in the 5'UTR of different HEV stains. The numbers in the brackets show the genotype designation. Click here for file
