# Supplementary figures and images for: Cloning of full genome sequence of hepatitis E virus of Shanghai swine isolate using RACE method
Source: Virol J. 2007 Oct 9;4:98. doi: 10.1186/1743-422X-4-98 (PMC2140057; doi:10.1186/1743-422X-4-98)

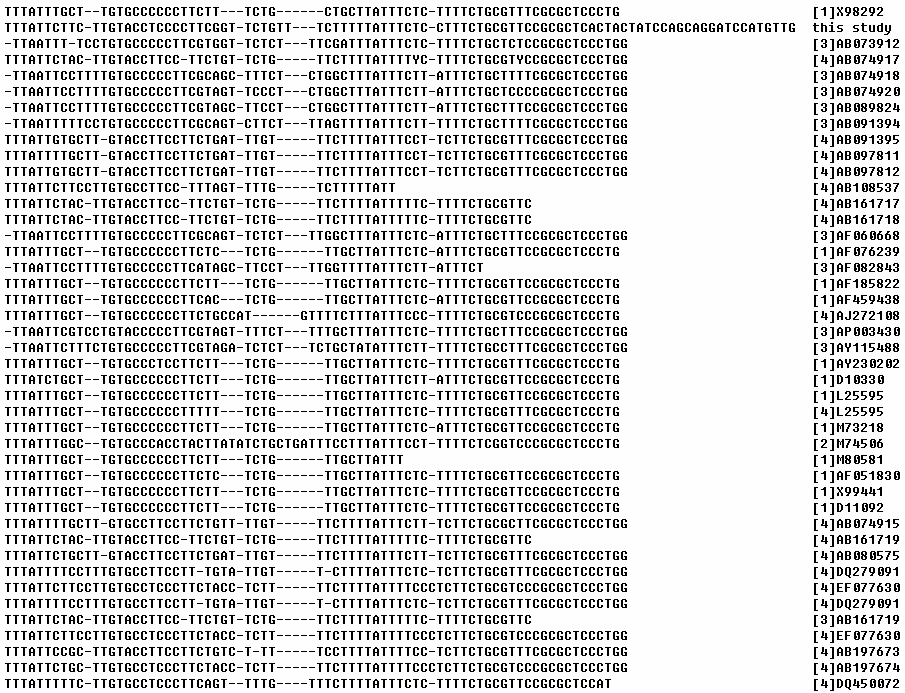

Supplement: Additional file 1 — Comparison of length in the 5'UTR of different HEV stains. The numbers in the brackets show the genotype designation. [file 1743-422X-4-98-S1.tiff]
